# Supplementary material for: Functional Cardiovascular Characterization of the Common Marmoset (Callithrix jacchus)
Source: Biology (Basel). 2023 Aug 11;12(8):1123. doi: 10.3390/biology12081123 (PMC10452209; doi:10.3390/biology12081123)
Supplement: Supplementary file 1 [file biology-12-01123-s001.zip › biology-2507293-SI.pdf]

biology

MDPI

Supplemental Material

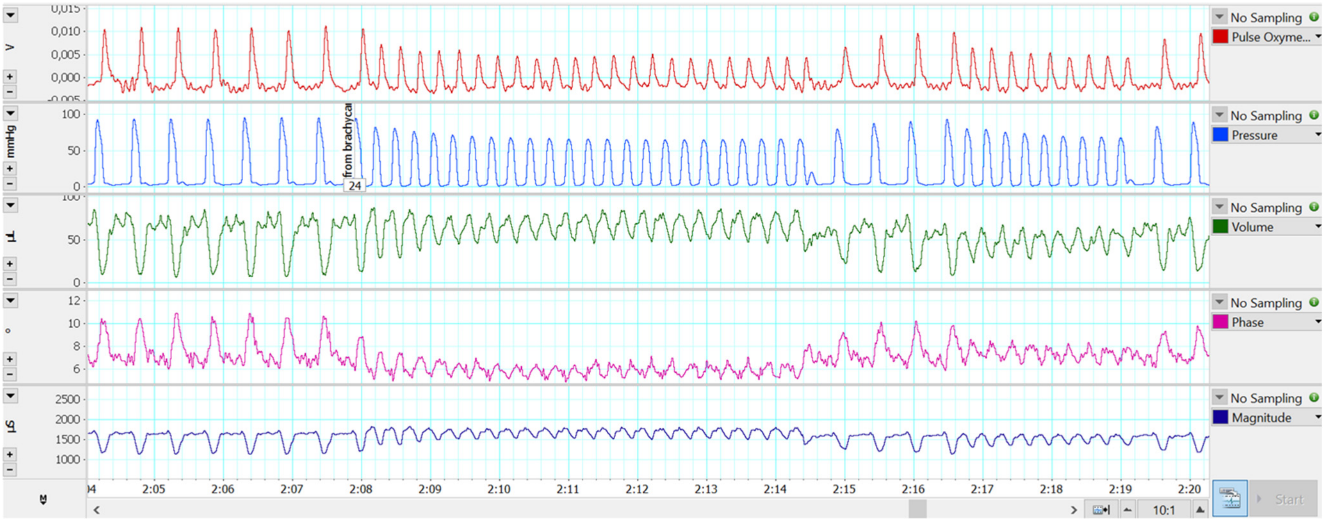

Figure S1. Representative tracings from LabChart Chart View of Animal 7 that was excluded due to arrhythmia.

	PV-loop	Echocardiography	MRI
**HR (bpm)**	202 ± 17	257 ± 17	230 ± 22
**ESV (μl)**	99 ± 23	87 ± 20	**368 ± 21***
**EDV (μl)**	325 ± 31	272 ± 30	**1000 ± 62***
**SV (μl)**	225 ± 31	185 ± 11	**631 ± 53***
**EF(%)**	70 ± 7	69 ± 3	63 ± 2
**CO (ml/min)**	45 ± 7	47 ± 2	**150 ± 23***

Table S1. Functional hemodynamic data acquired via PV-loop, Echocardiography and MRI of the same 5 animals.
HR = Heart Rate, ESV = End Systolic Volume, EDV = End Diastolic Volume, SV = Stroke Volume, EF = Ejection Fraction, CO = Cardiac Output
=*MRI data significantly different to PV-loop and Echocardiography ($p<0.05$) Data shown as mean ± SEM (n=5)

	Advantages	Disadvantages
**Marmoset**	- close phylogenetic relationship to humans [62, 63]<br>- relatively rapid reproduction with twin birth [7, 31, 64, 65]; twins exhibit bone marrow chimerism [7, 31, 66-68]<br>- commonly used for various biomedical research fields [7, 8, 69]<br>- relatively easy to handle [7, 8]<br>- least expensive NHP [31] with low zoonotic hazard [8, 31]<br>- used for CV research since the 80s [45, 70-72]<br>- similarities in heart anatomy and relative heart weight [19, 21]<br>- heart size more suitable compared to rats for angiographic investigation [70]<br>- spontaneous development of heart fibrosis, inflammatory cell infiltration and myocardial degeneration [41, 42]<br>- similar age-related changes in microvasculature [73]<br>- mean myocyte volume similar to man [40]<br>- similar age-related changes in cardiovascular system [22, 43], immunology [17] as well as neurological and motor system [74-77] making it suitable for aging research [11, 15, 62, 63, 78]<br>- spontaneous or induced models of obesity and diabetes [14]<br>- similar lipoprotein profile [79-81]<br>- good model to study renin-angiotensin system [82, 83]<br>- possibility for transgenic applications [63, 84]<br>-)similar ECG values(	- some aspects of the cardiovascular system have been studied (see Advantages), but heart function has never been comprehensively characterized<br>- rhesus macaques closer resemble the human situation [85]<br>- few researchers have experience with marmosets<br>- lower availability than small rodents<br>- relatively expensive/ complicated husbandry and breeding compared to rodents [38, 86]<br>- longer life cycle than rodents<br>- slight differences in cardiac anatomy [37] and myocyte structure [40] compared to human<br>- ethical considerations [36]<br>- heart rate differs from human<br>- difficulties in genetic modification [38]<br>- limited availability of cross-reacting antibodies [39]
**Pig**	- highest analogy of heart and blood vessel sizes [87]<br>- induced lesions more similar to human disease [88]<br>- collateralization enables total infarct by occluding left anterior descending artery [87]<br>- similarity in physiology, metabolism, genomics and proteomics to human [89, 90]	- high cost compared to rodents[38, 88]<br>- difficult handling [88]<br>- few genomic tools [88]<br>- most antibodies for human are not effective in pigs [90]

	- availability of various heart disease models and genetically established cardiomyopathy [89]<br>- valid for restenosis studies [88]<br>- genetically modified established model for human atherosclerosis [38]<br>- non-invasive techniques for heart catheterization [91]<br>- availability of monitoring systems in chronic models [91]<br>- availability of imaging-based study techniques [91]	
**Dog**	- docility and cognitive performance allow non-invasive and non-sedated assessments [92]<br>- shared genetic evolution with humans [92, 93]<br>- left ventricular function and volume similar to human [94]<br>- myosin heavy chain distribution similar to human [94]<br>- comparable electrophysiology [95]<br>- cardiac proteome similar to human [90]<br>- established disease models [96-98]<br>- genetic modifications possible [92]<br>- availability of monitoring systems in chronic models [91]<br>- availability of imaging-based study techniques [91]<br>- naturally occurring cardiomyopathies (summarized in [99])	- variation in coronary artery supply compared to humans as well as between individual dogs results in a complicated surgery setting for MI [87, 100, 101]<br>- differences in heart anatomy [102]<br>- relatively costly/ complicated housing and breeding [38, 90, 94]<br>- most antibodies for human are not effective in dogs [90]<br>- genetic modification not as easy as in small rodents [38, 103]<br>- complicated operation [38]<br>- ethical considerations due to companion animal status (in Europe)
**Mouse**	- large experience [38, 88]<br>- well-known genome, relative ease of genome manipulation [88]<br>- low cost [88, 94]<br>- short reproductive cycle [38, 88, 91, 94] allows for studies over short time period<br>- useful for noninvasive imaging [88]<br>- rapid development of atherosclerotic plaques in transgenic mice under specific diet [88]<br>- standardized feeding and housing conditions with low variability [38]	- only partial resemblance to humans [88]<br>- heart rate differs significantly from humans [94, 104]<br>- cardiac metabolism differs from human [105]<br>- action potential differs to the human one [94]<br>- different ratio of a- and ß-myosin heavy chains [94]<br>- atherosclerosis does not occur spontaneously in mice [106], more atherosclerotic than atherothrombosis model [88]<br>- very high level of blood lipids [88]
**Rat**	- easy to handle, available, low cost [88]<br>- short reproductive cycle [91]	- heart rate differs significantly from humans<br>- do not develop atheroma [88]

	Advantages	Disadvantages
	- established model of choice for decades in cardiovascular research [107]<br>- useful for restenosis analysis [88]	- modification of hemodynamic conditions in open-thorax models [91]
**Rabbit**	- established disease models [108, 109]<br>- inexpensive, easily available [88, 110]<br>- easy to handle, medium size [88]<br>- short reproductive cycle [111]<br>- mimics alteration of myocardial function observed in end stage failing human myocard [94]<br>- development of fibroartheroma lesion in atherosclerosis model [88]<br>- useful for restenosis models [88]<br>- WHHLMI strain develops myocardial infarction spontaneously [109]	- heart rate differs from humans [104]<br>- few genomic tools [88]<br>- differences in cardiac metabolism [103]<br>- need for high blood cholesterol levels for development of atherosclerosis [88]

Table S2. Selected advantages and disadvantages of different animal models relevant for cardiovascular research.

References

62. Fischer, K.E.; Austad, S.N. The development of small primate models for aging research. ILAR J. 2011, 52, 78–88. https://doi.org/10.1093/ilar.52.1.78.
63. Mattison, J.A.; Vaughan, K.L. An overview of nonhuman primates in aging research. Exp. Gerontol. 2017, 94, 41–45. https://doi.org/10.1016/j.exger.2016.12.005.
64. Abbott, D.H.; Hearn, J.P. Physical, hormonal and behavioural aspects of sexual development in the marmoset monkey, Callithrix jacchus. J. Reprod. Fertil. 1978, 53, 155–166. https://doi.org/10.1530/jrf.0.0530155.
65. Tardif, S.D.; Smucny, D.A.; Abbott, D.H.; Mansfield, K.; Schultz-Darken, N.; Yamamoto, M.E. Reproduction in captive common marmosets (Callithrix jacchus). Comp. Med. 2003, 53, 364–368.
66. Benirschke, K.; Anderson, J.M.; Brownhill, L.E. Marrow Chimerism in Marmosets. Science 1962, 138, 513–515. https://doi.org/10.1126/science.138.3539.513.
67. Benirschke, K.; Brownhill, L.E. Further observations on marrow chimerism in marmosets. Cytogenetics 1962, 1, 245–257. https://doi.org/10.1159/000129734.
68. Silva, M.O.M.; Armada, J.L.A.; Verona, C.E.S.; Heliodoro, G.; Nogueira, D.M. Cytogenetics and Molecular Genetic Analysis of Chimerism in Marmosets (Callithrix: Primates). An. Acad. Bras. Cienc. 2017, 89, 2793–2804. https://doi.org/10.1590/0001-3765201720170484.
69. Greenough, T.C.; Carville, A.; Coderre, J.; Somasundaran, M.; Sullivan, J.L.; Luzuriaga, K.; Mansfield, K. Pneumonitis and multi-organ system disease in common marmosets (Callithrix jacchus) infected with the severe acute respiratory syndrome-associated coronavirus. Am. J. Pathol. 2005, 167, 455–463. https://doi.org/10.1016/s0002-9440(10)62989-6.
70. Charnock, J.S.; McLennan, P.L.; McIntosh, G.H.; Barnden, L.R.; Buttfield, I.H. Radionuclide angiographic study of the influence of dietary lipid supplements on cardiac function in the marmoset (Callithrix jacchus). Cardiovasc. Res. 1987, 21, 369–376. https://doi.org/10.1093/cvr/21.5.369.
71. McMurchie, E.J.; Patten, G.S.; McLennan, P.L.; Charnock, J.S.; Nestel, P.J. The influence of dietary lipid supplementation on cardiac beta-adrenergic receptor adenylate cyclase activity in the marmoset monkey. Biochim. Biophys. Acta 1988, 937, 347–358. https://doi.org/10.1016/0005-2736(88)90257-x.

72. McMurchie, E.J.; Patten, G.S.; McLennan, P.L.; Charnock, J.S. A comparison of the properties of the cardiac beta-adrenergic receptor adenylyl cyclase system in the rat and the marmoset monkey. Comp. Biochem. Physiol. B 1987, 88, 989–998. https://doi.org/10.1016/0305-0491(87)90275-6.
73. Sobin, S.S.; Bernick, S.; Ballard, K.W. Histochemical characterization of the aging microvasculature in the human and other mammalian and non-mammalian vertebrates by the periodic acid-Schiff reaction. Mech. Ageing Dev. 1992, 63, 183–192. https://doi.org/10.1016/0047-6374(92)90064-k.
74. Geula, C.; Nagykery, N.; Wu, C.K. Amyloid-beta deposits in the cerebral cortex of the aged common marmoset (Callithrix jacchus): Incidence and chemical composition. Acta Neuropathol. 2002, 103, 48–58. https://doi.org/10.1007/s004010100429.
75. Berkovitz, B.K.; Pacy, J. Age changes in the cells of the intra-articular disc of the temporomandibular joints of rats and marmosets. Arch. Oral Biol. 2000, 45, 987–995. https://doi.org/10.1016/s0003-9969(00)00067-4.
76. Harada, T.; Tokuriki, M.; Tanioka, Y. Age-related changes in the brainstem auditory evoked potentials of the marmoset. Heart Res. 1999, 128, 119–124. https://doi.org/10.1016/s0378-5955(98)00201-9.
77. Black, A.; Lane, M.A. Nonhuman primate models of skeletal and reproductive aging. Gerontology 2002, 48, 72–80. https://doi.org/10.1159/000048930.
78. Ross, C.N.; Salmon, A.B. Aging research using the common marmoset: Focus on aging interventions. Nutr. Healthy Aging 2019, 5, 97–109. https://doi.org/10.3233/nha-180046.
79. Crook, D.; Weisgraber, K.H.; Boyles, J.K.; Mahley, R.W. Isolation and characterization of plasma lipoproteins of common marmoset monkey. Comparison of effects of control and atherogenic diets. Arteriosclerosis 1990, 10, 633–647. https://doi.org/10.1161/01.atv.10.4.633.
80. Abbey, M.; Clifton, P.M.; McMurchie, E.J.; McIntosh, G.H.; Nestel, P.J. Effect of a high fat/cholesterol diet with or without eicosapentaenoic acid on plasma lipids, lipoproteins and lipid transfer protein activity in the marmoset. Atherosclerosis 1990, 81, 163–174. https://doi.org/10.1016/0021-9150(90)90063-o.
81. Lima, V.L.; Sena, V.L.; Stewart, B.; Owen, J.S.; Dolphin, P.J. An evaluation of the marmoset Callithrix jacchus (sagüi) as an experimental model for the dyslipoproteinemia of human Schistosomiasis mansoni. Biochim. Biophys. Acta 1998, 1393, 235–243. https://doi.org/10.1016/s0005-2760(98)00076-9.
82. Michel, J.B.; Wood, J.; Hofbauer, K.; Corvol, P.; Menard, J. Blood pressure effects of renin inhibition by human renin antiserum in normotensive marmosets. Am. J. Physiol. 1984, 246, F309–F316. https://doi.org/10.1152/ajprenal.1984.246.3.F309.
83. Hiruma, M.; Kim, S.; Ikemoto, F.; Murakami, K.; Yamamoto, K. Fate of recombinant human renin administered exogenously to anesthetized monkeys. Hypertension 1988, 12, 317–323. https://doi.org/10.1161/01.hyp.12.3.317.
84. Drummer, C.; Vogt, E.J.; Heistermann, M.; Roshani, B.; Becker, T.; Mätz-Rensing, K.; Kues, W.A.; Kügler, S.; Behr, R. Generation and Breeding of EGFP-Transgenic Marmoset Monkeys: Cell Chimerism and Implications for Disease Modeling. Cells 2021, 10, 505. https://doi.org/10.3390/cells10030505.
85. Preuss, T.M. Critique of Pure Marmoset. Brain Behav. Evol. 2019, 93, 92–107. https://doi.org/10.1159/000500500.
86. Holtze, S.; Gorshkova, E.; Braude, S.; Cellerino, A.; Dammann, P.; Hildebrandt, T.B.; Hoeflich, A.; Hoffmann, S.; Koch, P.; Terzibasi Tozzini, E.; et al. Alternative Animal Models of Aging Research. Front. Mol. Biosci. 2021, 8, 660959. https://doi.org/10.3389/fmolb.2021.660959.
87. Swindle, M.M.; Makin, A.; Herron, A.J.; Clubb, F.J., Jr.; Frazier, K.S. Swine as models in biomedical research and toxicology testing. Vet. Pathol. 2012, 49, 344–356. https://doi.org/10.1177/0300985811402846.
88. Zaragoza, C.; Gomez-Guerrero, C.; Martin-Ventura, J.L.; Blanco-Colio, L.; Lavin, B.; Mallavia, B.; Tarin, C.; Mas, S.; Ortiz, A.; Egido, J. Animal models of cardiovascular diseases. J. Biomed. Biotechnol. 2011, 2011, 497841. https://doi.org/10.1155/2011/497841.
89. Hiroaki, O.; Kozue, M.; Hidetoshi, M. Large Animal Models in Cardiovascular Research. In Animal Models and Experimental Research in Medicine; Mahmut, K., Volkan, G., Abdulsamed, K., Eds.; IntechOpen: Rijeka, Croatia, 2022; p. Ch. 11.
90. Kooij, V.; Venkatraman, V.; Tra, J.; Kirk, J.A.; Rowell, J.; Blice-Baum, A.; Cammarato, A.; Van Eyk, J.E. Sizing up models of heart failure: Proteomics from flies to humans. Proteom. Clin. Appl. 2014, 8, 653–664. https://doi.org/10.1002/prca.201300123.
91. Chorro, F.J.; Such-Belenguer, L.; López-Merino, V. Animal Models of Cardiovascular Disease. Rev. Española Cardiol. (Engl. Ed.) 2009, 62, 69–84. https://doi.org/10.1016/S1885-5857(09)71516-6.

92. Barthélémy, I.; Hitte, C.; Tiret, L. The Dog Model in the Spotlight: Legacy of a Trustful Cooperation. J. Neuromuscul. Dis. 2019, 6, 421–451. https://doi.org/10.3233/jnd-190394.
93. Wang, G.D.; Zhai, W.; Yang, H.C.; Fan, R.X.; Cao, X.; Zhong, L.; Wang, L.; Liu, F.; Wu, H.; Cheng, L.G.; et al. The genomics of selection in dogs and the parallel evolution between dogs and humans. Nat. Commun. 2013, 4, 1860. https://doi.org/10.1038/ncomms2814.
94. Hasenfuss, G. Animal models of human cardiovascular disease, heart failure and hypertrophy. Cardiovasc. Res. 1998, 39, 60–76. https://doi.org/10.1016/S0008-6363(98)00110-2.
95. Kaese, S.; Frommeyer, G.; Verheule, S.; van Loon, G.; Gehrmann, J.; Breithardt, G.; Eckardt, L. The ECG in cardiovascular-relevant animal models of electrophysiology. Herzschrittmacherther. Elektrophysiol. 2013, 24, 84–91. https://doi.org/10.1007/s00399-013-0260-z.
96. Lavine, S.J. Effect of changes in contractility on the index of myocardial performance in the dysfunctional left ventricle. Cardiovasc. Ultrasound 2006, 4, 45. https://doi.org/10.1186/1476-7120-4-45.
97. Loen, V.; Vos, M.A.; van der Heyden, M.A.G. The canine chronic atrioventricular block model in cardiovascular preclinical drug research. Br. J. Pharmacol. 2022, 179, 859–881. https://doi.org/10.1111/bph.15436.
98. Powers, J.C.; Recchia, F. Canine Model of Pacing-Induced Heart Failure. Methods Mol. Biol. 2018, 1816, 309–325. https://doi.org/10.1007/978-1-4939-8597-5_24.
99. Duncker, D.J.; Bakkers, J.; Brundel, B.J.; Robbins, J.; Tardiff, J.C.; Carrier, L. Animal and in silico models for the study of sarcomeric cardiomyopathies. Cardiovasc. Res. 2015, 105, 439–448. https://doi.org/10.1093/cvr/cvv006.
100. Lowe, J.E.; Reimer, K.A.; Jennings, R.B. Experimental infarct size as a function of the amount of myocardium at risk. Am. J. Pathol. 1978, 90, 363–379.
101. Khan, M.S.; Smego, D.; Ishidoya, Y.; Hirahara, A.M.; Offei, E.; Ruiz Castillo, M.S.; Gharbia, O.; Li, H.; Palatinus, J.A.; Krueger, L.; et al. A canine model of chronic ischemic heart failure. Am. J. Physiol. Heart Circ. Physiol. 2023, 324, H751–H761. https://doi.org/10.1152/ajpheart.00647.2022.
102. Chevènement, O.; Borenstein, N.; Kieval, R.; Fiette, L.; Aujard, F. Animal model considerations to evaluate prosthetic tricuspid valve implants. Ann. Anat. 2021, 234, 151625. https://doi.org/10.1016/j.aanat.2020.151625.
103. Milani-Nejad, N.; Janssen, P.M. Small and large animal models in cardiac contraction research: Advantages and disadvantages. Pharmacol. Ther. 2014, 141, 235–249. https://doi.org/10.1016/j.pharmthera.2013.10.007.
104. Hornyik, T.; Rieder, M.; Castiglione, A.; Major, P.; Baczko, I.; Brunner, M.; Koren, G.; Odening, K.E. Transgenic rabbit models for cardiac disease research. Br. J. Pharmacol. 2022, 179, 938–957. https://doi.org/10.1111/bph.15484.
105. Ginis, I.; Luo, Y.; Miura, T.; Thies, S.; Brandenberger, R.; Gerecht-Nir, S.; Amit, M.; Hoke, A.; Carpenter, M.K.; Itskovitz-Eldor, J.; et al. Differences between human and mouse embryonic stem cells. Dev. Biol. 2004, 269, 360–380. https://doi.org/10.1016/j.ydbio.2003.12.034.
106. Emini Veseli, B.; Perrotta, P.; De Meyer, G.R.A.; Roth, L.; Van der Donckt, C.; Martinet, W.; De Meyer, G.R.Y. Animal models of atherosclerosis. Eur. J. Pharmacol. 2017, 816, 3–13. https://doi.org/10.1016/j.ejphar.2017.05.010.
107. Bader, M. Rat Models of Cardiovascular Diseases. In Rat Genomics: Methods and Protocols; Anegon, I., Ed.; Humana Press: Totowa, NJ, USA, 2010; pp. 403–414. https://doi.org/10.1007/978-1-60327-389-3_27.
108. González, G.E.; Seropian, I.M.; Krieger, M.L.; Palleiro, J.; Lopez Verrilli, M.A.; Gironacci, M.M.; Cavallero, S.; Wilensky, L.; Tomasi, V.H.; Gelpi, R.J.; et al. Effect of early versus late AT(1) receptor blockade with losartan on postmyocardial infarction ventricular remodeling in rabbits. Am. J. Physiol. Heart Circ. Physiol. 2009, 297, H375–H386. https://doi.org/10.1152/ajpheart.00498.2007.
109. Shiomi, M.; Ito, T.; Yamada, S.; Kawashima, S.; Fan, J. Development of an animal model for spontaneous myocardial infarction (WHHLMI rabbit). Arterioscler. Thromb. Vasc. Biol. 2003, 23, 1239–1244. https://doi.org/10.1161/01.Atv.0000075947.28567.50.
110. Chobanian, A.V.; Arquilla, E.R.; Clarkson, T.B.; Eder, H.A.; Howard, C.F., Jr.; Regan, T.J.; Williamson, J.R. Cardiovascular complications. Diabetes 1982, 31, 54–64. https://doi.org/10.2337/diab.31.1.s54.
111. Calasans-Maia, M.D.; Monteiro, M.L.; Ascoli, F.O.; Granjeiro, J.M. The rabbit as an animal model for experimental surgery. Acta Cir. Bras. 2009, 24, 325–328. https://doi.org/10.1590/s0102-86502009000400014.
